# Increased acetylation of Peroxiredoxin1 by HDAC6 inhibition leads to recovery of Aβ-induced impaired axonal transport

**DOI:** 10.1186/s13024-017-0164-1

**Published:** 2017-02-28

**Authors:** Heesun Choi, Haeng Jun Kim, Jisoo Kim, Soohyun Kim, Jinhee Yang, Wonik Lee, Yeonju Park, Seung Jae Hyeon, Dong-Sup Lee, Hoon Ryu, Junho Chung, Inhee Mook-Jung

**Affiliations:** 1 Department of Biochemistry and Biomedical Sciences, Seoul National University, College of Medicine, Seoul, Korea; 2 Department of Biochemistry and Molecular Biology, Seoul National University, College of Medicine, Seoul, Korea; 3 Cancer Research Institute, Seoul National University College of Medicine, Seoul, Korea; 4 Department of Biomedical Sciences, Laboratory of Immunology and Cancer Biology, Seoul National University College of Medicine, Seoul, Korea; 5 VA Boston Healthcare System, Boston University Alzheimer’s Disease Center, and Department of Neurology, Boston University School of Medicine, Boston, MA02130 USA; 6 Center for Neuromedicine, Brain Science Institute, Korea Institute of Science and Technology, Seoul, Korea

**Keywords:** Alzheimer’s disease, Histone deacetylase 6, Peroxiredoxin1, Axonal transport, Oxidative stress, Reactive oxygen species

## Abstract

**Background:**

Reduction or inhibition of histone deacetylase 6 (HDAC6) has been shown to rescue memory in mouse models of Alzheimer’s disease (AD) and is recently being considered a possible therapeutic strategy. However, the restoring mechanism of HDAC6 inhibition has not been fully understood.

**Methods and results:**

Here, we found that an anti-oxidant protein Peroxdiredoxin1 (Prx1), a substrate of HDAC6, malfunctions in Aβ treated cells, the brains of 5xFAD AD model mice and AD patients. Malfunctioning Prx1, caused by reduced Prx1 acetylation levels, was recovered by HDAC6 inhibition. Increasing acetylation levels of Prx1 by HDAC6 inhibition recovered elevated reactive oxygen species (ROS) levels, elevated Ca^2+^ levels and impaired mitochondrial axonal transport, sequentially, even in the presence of Aβ. Prx1 mutant studies on the K197 site for an acetylation mimic or silencing mutation support the results showing that HDAC6 inhibitor restores Aβ-induced disruption of ROS, Ca^2+^ and axonal transport.

**Conclusions:**

Taken together, increasing acetylation of Prx1 by HDAC6 inhibition has several beneficial effects in AD pathology. Here, we present the novel mechanism by which elevated acetylation of Prx1 rescues mitochondrial axonal transport impaired by Aβ. Therefore, our results suggest that modulation of Prx1 acetylation by HDAC6 inhibition has great therapeutic potential for AD and has further therapeutic possibilities for other neurodegenerative diseases as well.

**Electronic supplementary material:**

The online version of this article (doi:10.1186/s13024-017-0164-1) contains supplementary material, which is available to authorized users.

## Background

Alzheimer’s disease (AD) is the most common neurodegenerative disease that leads to cognitive impairment. The major pathological features of AD are extracellular accumulation of beta-amyloid (Aβ), called senile plaque, and intracellular neurofibrillary tangles which are composed of hyperphosphorylated tau [1, 2]. There are several well-known cytotoxic effects and molecular changes of Aβ. Aβ disrupts axonal transport by dysregulating microtubule stability and affinity between motor or adaptor proteins and microtubule or cargos [3, 4]. Axonal transport is important for neuronal function and cell viability [5–7]. Oxidative stress, which is caused by increased reactive oxygen species (ROS), and excessive cytosolic Ca^2+^ are induced by Aβ [1]. ROS oxidize lipids, proteins and nucleic acids which are essential for normal cellular function, which leads to increased membrane permeability to Ca^2+^, mitochondrial damage and apoptosis [8–11]. Excessive Ca^2+^ is involved in excitotoxicity which also contributes to neuronal cell death [12, 13]. In addition, ROS and Ca^2+^ damage to mitochondrial axonal transport is mediated by reduced affinity between Miro and kinesin [14, 15].

One of the molecular changes in AD include increased levels and activity of histone deacetylase 6 (HDAC6) in the brains of AD patients [16, 17]. HDAC6 is localized in the cytoplasm and deacetylates cytosolic proteins such as α-tubulin and Peroxiredoxin1 (Prx1) [18–20]. Some researchers have shown that reducing or inhibiting HDAC6 in AD model mice resulted in improved memory [21–23]. In addition, Aβ-induced impairment of mitochondrial axonal transport was rescued by HDAC6 inhibitor in primary neurons [24]. Even though HDAC6 has great possibility as an AD therapeutic target, the restoring mechanisms of HDAC6 inhibition are not fully understood.

Here we found one possible mechanism which is mediated by Prx1. A previous report has shown that increasing Prx1 acetylation elevates its reducing activity [19]. Since Prx1 is an HDAC6 substrate, increased HDAC6 activity may cause poor reducing activity of Prx1. Therefore, we studied whether HDAC6 inhibition can regulate ROS level through acetylation of Prx1. Furthermore, we tried to elucidate whether restored axonal transport by HDAC6 inhibitor is mediated by Prx1. Together, we propose that increased acetylation of Prx1 by HDAC6 inhibition leads to recovery of Aβ-induced pathology such as elevation of ROS and Ca^2+^ and impaired axonal transport, making HDAC6 a possible therapeutic target for AD.

## Methods

### Human brain samples

Neuropathological processing of normal and AD human brain samples followed the procedures previously established for the Boston University Alzheimer’s Disease Center (BUADC). Entorhinal cortex and hippocampal regions were used for experiments. Detailed information of brain tissues is described in Additional file 1. In all cases where AD was diagnosed at autopsy, AD was stated as the cause of death. AD subjects had no evidence of other neurological disease based on neuropathological examination. Next of kin provided informed consent for participation and brain donation. The study was performed in accordance with principles of human subject protection in the Declaration of Helsinki. This study was reviewed by the Boston University School of Medicine Institutional Review Board and was approved as exempt because the study involves only tissue collected from post-mortem, and consequently not classified as human subjects.

### Animals and intraperitoneal (i.p.) injection

Six-month-old 5xFAD mice (Tg6799; B6SJL-Tg (APPSwFlLon, PSEN* M146L*L286V) 6799Vas/J, stock number 006554, Jackson Labs, Bar Harbor, ME, USA) overexpressing human amyloid precursor protein 695 with three mutations (Swedish, Florida and London) and human presenilin 1 with two mutations (M146L and L286V) under transcriptional control of the murine Thy-1 promoter and wild type littermate (B6/SJL) were used for brain tissue analysis after Tubastatin A (TBA) injection. TBA (100 mg/kg) or saline was administrated daily by i.p. injection for 4 weeks. Animals were treated and maintained in accordance with the Animal Care and Use Guidelines of Seoul National University, Seoul, Korea.

### Cell culture and transfection

HT22 cells were cultured in Dulbecco’s modified Eagles medium (DMEM; HyClone, USA) supplemented with 10% fetal bovine serum (Hyclone, USA) and 1% penicillin/streptomycin (Sigma, USA) at 37 °C under 5% CO_2_. Primary neuronal were previously described [25]. Briefly, primary hippocampal neurons were obtained from the brain tissue of Sprague–Dawley rat embryos (E18) (KOATECH, Korea). Brains were incubated in Hank’s Balanced Salt Solution (HBSS; WelGENE, Korea) with 0.05% trypsin (Gibco, USA) for 20 min at 37 °C. Neurons dissociated in NeuroBasal medium (Gibco, USA) supplemented with B27 (Gibco, USA) and penicillin/streptomycin were plated on poly-D-lysine (Sigma, USA) coated dishes or microfluidic chambers for immunoprecipitation or mitochondrial axonal transport analysis, respectively. Half of the culture medium was replaced with fresh medium every 3 days for plates and every day for microfluidic chambers. For transfection, constructs were mixed with Lipofectamine LTX (Invitrogen, USA) in Opti-MEM (Gibco, USA) for HT22 cells and Lipofectamine 2000 (Invitrogen, USA) in fresh neuronal culture medium for primary neurons.

### DNA constructs, reagents and antibodies

Three Flag-tagged Prx1 constructs – Prx1-WT-Flag, Prx1-K197Q-Flag and Prx1-K197R-Flag – and another three non-tagged vectors that express GFP separately – Prx1-WT, Prx1-K197Q and Prx1-K197R - were used. Prx1-WT-Flag construct based on pCR3.1 vector was kindly gifted by Dr. Sang Won Kang (Ewha Womans University, Seoul, Korea). Prx1-K197Q-Flag and Prx1-K197R-Flag vectors were generated by point mutation from Prx1-WT-Flag vector using site-directed mutagenesis kit (Enzynomics, Korea) according to manufacturer’s instructions. Three non-tagged, separately GFP-expressing Prx1 vectors were constructed from Flag-tagged Prx1 vectors by BamHI and NotI restriction and insertion into pBI-CMV2 vector (Clontech, USA) which has two identical independently expressed promoters, one of which expresses AcGFP. For mitochondrial labeling, pDsRed2-Mito (Clontech, USA) were used. Tubastatin A (TBA) was purchased from Sigma (USA) and U-chem (Korea). N-acetyl cysteine (NAC), Trolox (Sigma, USA) and BAPTA-AM (ThermoFisher Scientific, USA), Aβ_1–42_ peptide (American peptide, USA and Bachem, Switzerland) were used. Anti-Flag, acetyl-tubulin and β-actin antibodies were purchased from Sigma (USA). Antibodies against Prx1 (CST, USA), 4-HNE, 8-OHdG (Abcam, UK) and Tom2 (SantaCruz, USA) were also used. Acetyl-Prx1 antibodies were generated from chicken using BSA-conjugated synthetic peptide (Peptron Inc., Korea) with a sequence of SKEYFSK(Ac)QK, C-terminal of Prx1. For antibody selection, four rounds of bio-panning were performed as described previously using magnetic bead conjugated with BSA-acetylated Prx1 peptide after removing non-acetylated Prx1 binders using BSA-non-acetylated Prx1 peptide (BSA-CGGGSSKEYFSKQK) [26]. Affinity chromatography using Protein A agarose beads (Repligen 16 Corp., USA) was then used to purify acetyl-Prx1 antibodies (clone name: R2-31) [27].

### Preparation of Aβ

Aβ_1–42_ peptide (American peptide, USA and Bachem, Switzerland) was prepared as previously described [25]. In brief, Aβ_1–42_ peptide was dissolved in 1,1,1,3,3,3-hexafluoro-2-propanol (Sigma, USA) and lyophilized in a Speedvac (Labconco, USA). The dry peptide was dissolved in anhydrous dimethyl sulfoxide (Sigma, USA) at a final concentration of 1 mM and diluted in DMEM or cell culture medium. During treating to cells, most of Aβ consisted predominantly of oligomers and some monomers [24].

### Immunoprecipitation and western blotting

Cells were lysed in 1% Triton X-100 in TBS buffer (50 mM Tris HCl, 150 mM NaCl, pH 7.4) containing protease inhibitor cocktail, phenyl-methylsulfonyl fluoride (PMSF) (Sigma, USA) and TBA for HDAC6 inhibition. Flag-tagged Prx1 was immunoprecipitated using anti-Flag M2 magnetic beads (Sigma, USA) and eluted by competition with 3xFlag peptide (Sigma, USA) according to manufacturer’s instructions. Elutes were mixed with SDS-PAGE sample buffer, and boiled at 95 °C for 3 min. For immunoprecipitation of endogenous Prx1, anti-Prx1 antibodies and protein A/G agarose beads (SantaCruz, USA) were crosslinked by BS3 (ThermoFisher Scientific, USA) according to manufacturer’s instructions and then incubated with cell lysates overnight at 4 °C. Precipitates were eluted with SDS-PAGE sample buffer by boiling at 95 °C for 3 min. Both boiled elutes and equal amounts of input samples were separated via SDS-PAGE and transferred to polyvinylidene difluoride (PVDF) membranes. Membranes were blocked with 5% skim milk (Bioworld, USA) and probe with antibodies against indicated proteins. Western blotting process has been also described in a previous report [24].

### DCFDA assay

For ROS measurement, cells were treated with 1 μM of cell-permeant 2′, 7′-dichlorodihydrofluorescein diacetate (H_2_DCFDA, mentioned DCFDA in this paper) (Invitrogen, USA) in DMEM. After 1 h incubation at 37 °C, DCFDA was changed with DMEM. Fluorescent signals were captured using fluorescence microscope (Olympus, Japan) or CellInsight (Thermo Scientific, USA). Images obtained from fluorescence microscope were analyzed using Image J (NIH) and images from CellInsight using its software.

### Fluo-4 assay

For measuring Ca^2+^ concentration, cells were incubated with fluo-4 (Invitrogen, USA) for 1 h at 37 °C. After changing medium to DMEM, fluorescent signals were captured using fluorescence microscope or CellInsight. Images obtained from fluorescence microscope were analyzed using Image J (NIH) and from CellInsight using its software.

### Mitochondrial axonal transport analysis

Fabrication of microfluidic chambers and analysis of mitochondrial axonal transport were described in the reference [24]. In brief, neurons cultured in microfluidic chambers were transfected with pDsRed2-Mito at day in vitro (DIV) 7 or 8 to visualize mitochondria. Prx1 constructs, which express AcGFP, were simultaneously transfected with pDsRed2-Mito. After treatment with indicated compounds, live cells were time-lapse imaged using Olympus IX81 microscope (Japan) equipped with a Cool SNAP HQ2 CCD camera (Photometrics, USA), controlled by MetaMorph Software (Universal Imaging, USA), for 2 min, with a 1 s interval in an incubating equipment (Live cell instrument, Korea) which maintains 37 °C and 5% CO_2_. Movies were processed using MetaMorph. Mitochondrial movement in axons were analyzed by using Image J installed with multiple kymograph plugins (by J. Rietdorf and A. Seitz).

### Immunohistochemistry

Mice were anesthetized with a mixture of Zoletil 50 (Virbac, France) and Rompun (Bayer, USA) solution (3:1 ratio, 1 ml/kg, i.p.) and were transcardially perfused with phosphate buffered-saline (PBS). The hemisphere of the brain was dissected and incubated in 4% paraformaldehyde (PFA) for 36 h, followed by 30% sucrose for 72 h at 4 °C. Serial 30-μm-thick coronal tissue sections were made using freezing microtome (Leica, USA). For 4-HNE and 8-OHdG immunostaining using 3, 3′-diaminobenzidine (DAB), free-floating sections were incubated overnight at 4 °C with anti-4-HNE (1:200) and 8-OHdG (1:400) antibodies diluted in PBS containing 0.3% Triton X-100, 0.05% bovine serum albumin (BSA) and normal horse serum. Sections were then treated with 3% H_2_O_2_ in PBS for 30 min at room temperature (RT) to quench the activity of endogenous peroxidase, followed by incubation with biotinylated secondary antibodies (1:200; Vector Laboratories, USA) for 2 h at RT and then with an avidin-biotin complex (Vector Laboratories, USA) for 1 h at RT. Immunoreactivity was visualized by DAB in 0.05 M Tris-buffered saline (pH 7.6). Finally, sections were mounted on poly-L-lysine (Sigma, USA) coated Histobond glass slides (Marienfeld, Germany), air-dried overnight, serial ethanol dehydrated and coverslipped with Permount (Fisher Scientific, USA). For fluorescent staining with anti-acetyl-Prx1 (1:50) and Tom20 (1:200), sections were incubated overnight at 4 °C with indicated antibodies diluted in PBS containing 1% Triton X-100 and normal horse serum, followed by incubation with Alexa Fluor 488 and 594 secondary antibodies (ThermoFisher Scientific, USA) for 1 h at RT. Sections were washed with PBS every incubation step. For acetyl-Prx1 staining of human brain tissues using DAB, paraffin-embedded tissue sections were deparaffinized in 55 °C dry-oven for overnight. Rehydrated and endogenous peroxidase quenched tissue sections were incubated with anti-acetyl-Prx1 antibody (1:100) at 4 °C for overnight and the rest of procedures were equal to be described above. The images were acquired using Olympus FSX 100 (Olympus, Japan) or confocal microscope (Carl Zeiss, Germany) for DAB stained sections or fluorescent labeling sections, respectively. Image J was used for quantifying immunoreactivity.

### Statistical analysis

Data were analyzed by two-way analysis of variance (ANOVA) or one-way ANOVA with Bonferroni post-hoc tests. All data were shown as mean ± SEM.

## Results

### Acetylation level of Prx1 was decreased in the brains of AD patients and acetylation of Prx1 is regulated by both Aβ and HDAC6

**Fig. 1:**
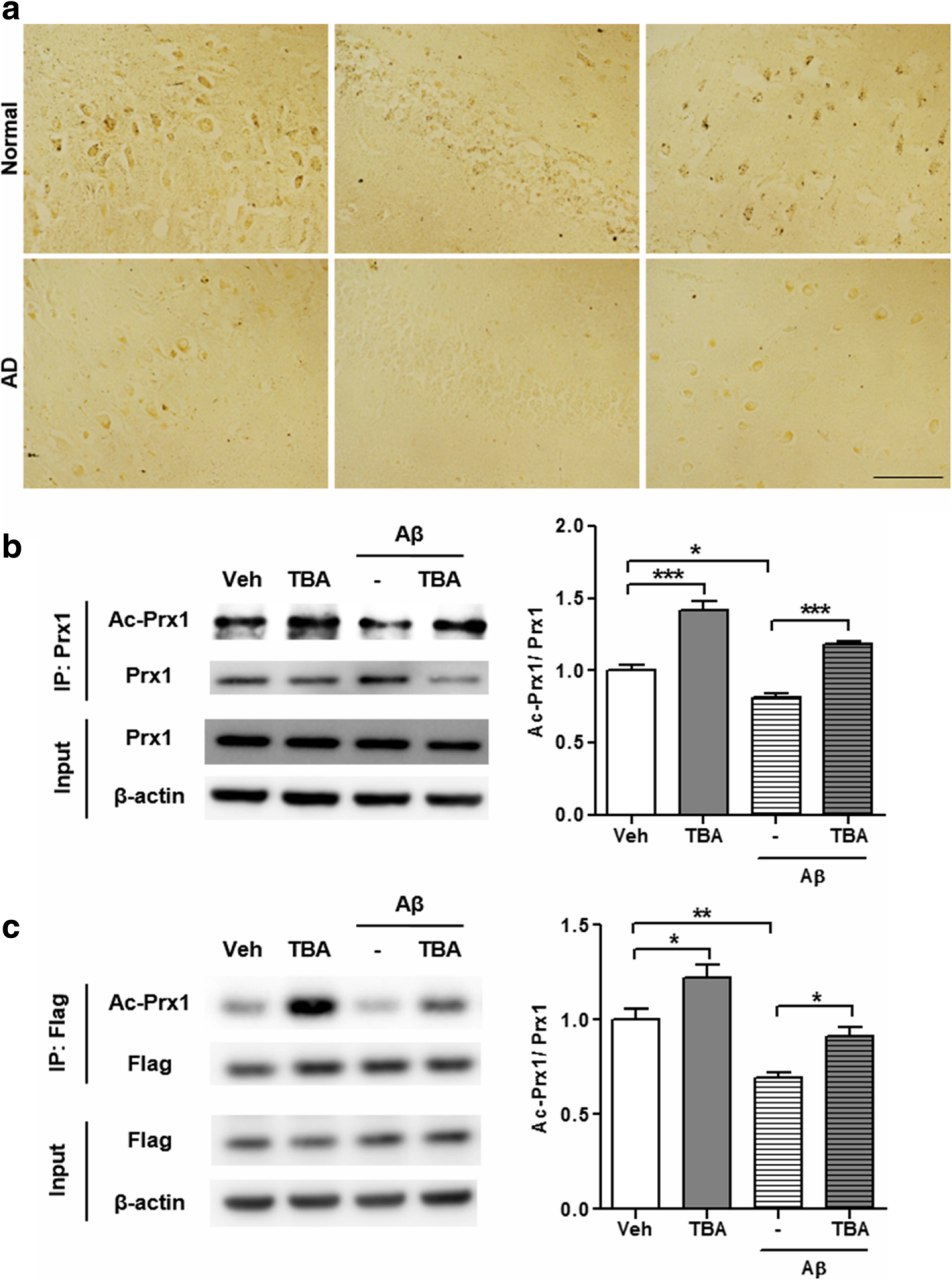
Acetyl-Prx1 is decreased in AD patients’ brains, and Aβ and HDAC6 inhibitor modulate Prx1 acetylation. **a** Representative images of acetyl-Prx1 level in human brain samples. *Top* row shows representative images of age- and sex-matched normal control brains and *bottom* row shows AD brains. Scale bar: 100 μm. **b** Acetylation level of Prx1 in primary hippocampal neurons. Primary hippocampal neurons were treated with Aβ (2 μM, 24 h) and then co-treated with TBA (1 μM, 3 h). Endogenous Prx1 was immunoprecipitated by anti-Prx1 antibody which was cross-linked to protein A/G coated agarose beads and probed by indicated antibodies. *Left* panel shows immunoblot images and *right* panel shows quantification of acetylation level of Prx1 which normalized by immunoprecipitated total Prx1 (*n* = 4, independent experiments). **c** Acetylation level of Prx1 in HT22 cells. Prx1-WT-Flag transfected HT22 cells were treated with Aβ (2 μM, 24 h) and then co-treated with TBA (0.5 μM, 3 h). Prx1-Flag was immunoprecipitated by anti-Flag M2 magnetic beads and probed by indicated antibodies. *Left* panel shows immunoblot images and right panel shows quantification of acetylation level of Prx1 which normalized by immunoprecipitated total Prx1 (*n* = 4, independent experiments). Data are presented as mean ± SEM. *P < 0.05, **P < 0.01, ***P < 0.001 (two-way ANOVA, Bonferroni post-hoc test)

Since it was reported that the level and activity of HDAC6 were increased in AD [16, 17] and Prx1 is one of substrates of HDAC6 [19], we hypothesized that acetylation of Prx1 is reduced in AD conditions. First, we confirmed this hypothesis in human brain samples (Fig. 1a). As expected, Prx1 acetylation was decreased in the hippocampus and entorhinal cortex of AD patients compared to age- and sex-matched normal controls. This result suggests that upregulated HDAC6 in AD brains could affect Prx1 acetylation level, and reduced acetylation level of Prx1 is one of pathological features of AD brains. To determine whether acetylation of Prx1 is affected by Aβ and HDAC6, we used primary hippocampal neurons and HT22 cell line. Endogenous Prx1 in primary hippocampal neurons showed that acetylation of Prx1 was reduced by Aβ and recovered by Tubastatin A (TBA), an HDAC6 inhibitor (Fig. 1b). Similar results were shown in HT22 cells. In HT22 cells, Aβ decreased the level of Prx1 acetylation, however, TBA treatment increased the level of Prx1 acetylation even in the presence of Aβ (Fig. 1c). These results suggest that acetylation of Prx1 might be one of the crucial factors that modulate AD pathology and that HDAC6 is involved in these processes. To confirm the deacetylation site on Prx1 by HDAC6, antibodies against acetyl Prx1 at lysine (K) 197 residue were developed and validated by immunoprecipitation of a Prx1-WT-Flag and a Prx1-K197R-Flag which was not acetylated at K197 by substituting K to arginine (R) (see Additional file 2). To increase acetylation at K197 of Prx1, the Prx1-WT-Flag transfected HT22 cells were treated with TBA. It can be confirmed that the acetyl Prx1 antibody (R2-31) specifically detects acetylation of Prx1 at K197 because there was no signal in the Prx1-K197R-Flag, but there was in the Prx1-WT-Flag. In addition, a stronger signal appeared in the TBA-treated Prx1-WT-Flag than the non-treated one. These findings show that acetylation of Prx1 is regulated by both Aβ and HDAC6.

### ROS and Ca^2+^ are regulated by HDAC6 inhibitor

**Fig. 2:**
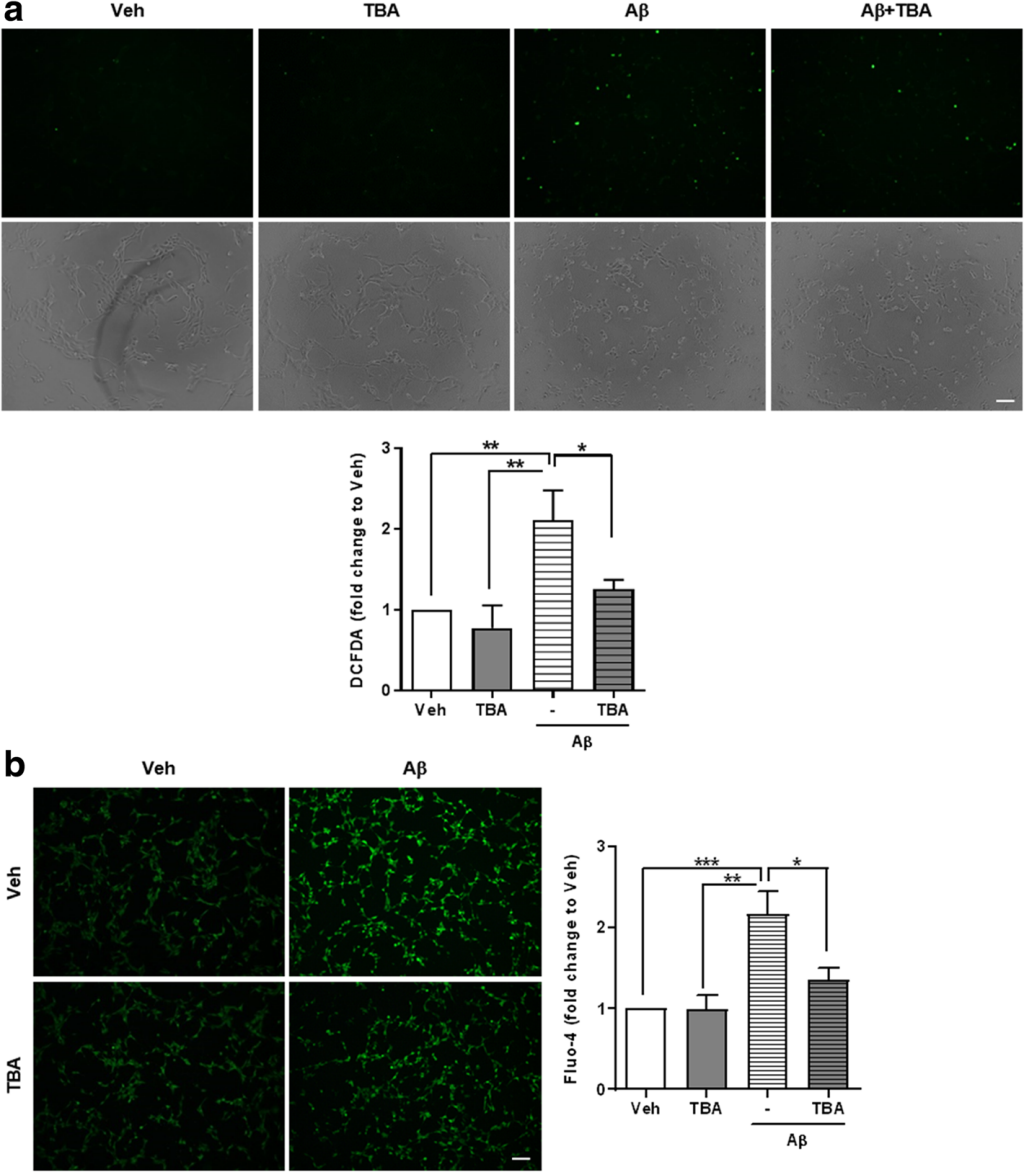
HDAC6 inhibition decreases Aβ-induced ROS and Ca^2+^. HT22 cells were treated with TBA (0.5 μM, 3 h) after being treated with Aβ (2 μM, 24 h). **a** ROS level was measured by DCFDA assay in HT22 cells. *Upper* panel shows representative DCFDA signals (*top* row) and bright field images (*bottom* row). *Lower* panel shows quantitative graph of fluorescent intensity (*n* = 5, independent experiments). **b** Ca^2+^ level was measured by Fluo-4 assay in HT22 cells. *Left* panel is representative images and *right* panel is quantification of fluorescent intensity (*n* = 5, independent experiments). Data are presented as mean ± SEM. *P < 0.05, **P < 0.01, ***P < 0.001 (two-way ANOVA, Bonferroni post-hoc test). Scale bar: 100 μm

Since increased acetylation of Prx1 shows more effective antioxidant activity [19], we tested whether acetylated Prx1 reduces ROS levels in HT22 cells. To prove it, we performed a DCFDA assay to measure ROS level in HT22 cells which were treated with Aβ with or without TBA. The fluorescence signal of DCFDA was increased in the Aβ treated group compared to veh. However, in the Aβ and TBA co-treatment group, the signal was decreased compared to the Aβ treated group (Fig. 2a). This indicates that the HDAC6 inhibitor can reduce the Aβ-induced elevated ROS level. Several reports showed that excessive ROS elevates cytoplasmic Ca^2+^ level through IP3R or RyR [28–30]. Since Aβ is also known to increase intracellular Ca^2+^ level, we measured Ca^2+^ level using Fluo-4 assay at the same condition as above. Similar with ROS level, Ca^2+^ level was increased by Aβ and rescued by TBA in the presence of Aβ (Fig. 2b). In addition, the results of TBA pretreatment before Aβ were also showed recovered Aβ-induced ROS and Ca^2+^ levels by TBA, which were similar to those of Fig. 2 - posttreatment of TBA (Additional file 3). These results provide evidence that increased acetylation of Prx1 by HDAC6 inhibitor might regulate ROS as well as Ca^2+^ levels in the presence of Aβ.

### Aβ-induced ROS regulates intracellular Ca^2+^ level

**Fig. 3:**
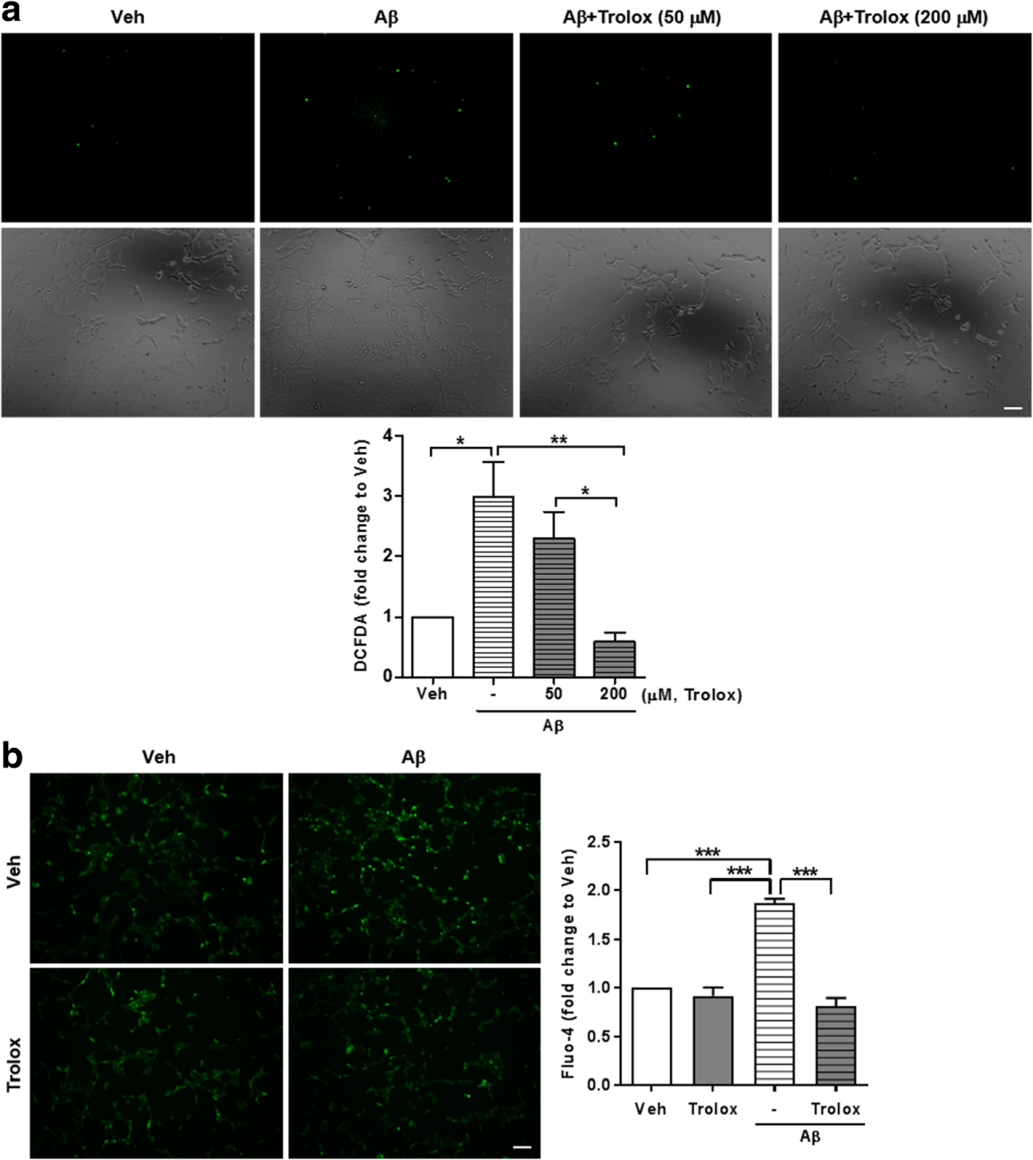
Aβ-induced ROS regulates intracellular calcium level. **a** Aβ-induced ROS was decreased by 200 μM trolox. HT22 cells were pretreated with indicated concentration of trolox for 1 h before incubation with 2 μM Aβ (24 h). *Upper* panel is representative images of DCFDA signals (*top* row) and bright field (*bottom* row) to measure ROS level in HT22 cells and *lower* penal is quantitative graph (*n* = 4, independent experiments). The results were shown as mean ± SEM. *P < 0.05, **P < 0.01 (one-way ANOVA, Bonferroni post-hoc test) **b** Reduction of Aβ-induced ROS level by trolox can decrease Ca^2+^ level. HT22 cells were pretreated with 200 μM trolox for 1 h before incubation with 2 μM Aβ (24 h). *Left* panel is representative images of Fluo-4 assay to measure Ca^2+^ level in HT22 cells and *right* panel is quantitative graph (*n* = 5, independent experiments). The results were shown as mean ± SEM. ***P < 0.001 (two-way ANOVA, Bonferroni post-hoc test). Scale bar: 100 μm

To explore whether ROS can regulate Ca^2+^ level in the presence of Aβ, we treated trolox and N-acetyl cysteine (NAC) with Aβ to HT22 cells. Both trolox and NAC, which is an analog of vitamin E and a precursor to glutathione, respectively, were ROS inhibitors. 200 μM of trolox was enough to suppress ROS when co-treated with Aβ in HT22 cells (Fig. 3a). Under this condition (200 μM trolox), where the ROS level is similar to veh even in the presence of Aβ, the Ca^2+^ level measured by Fluo-4 assay was also decreased compared to the Aβ-treated group (Fig. 3b). NAC, which is a different species of ROS inhibitor, had similar effects on Aβ-induced ROS and Ca^2+^ levels (Additional file 4). These results suggested that ROS could regulate Ca^2+^ level. In addition, the reverse direction, in which Ca^2+^ induces ROS, was examined in the presence of Aβ. HT22 cells were treated with BAPTA-AM, which is an intracellular Ca^2+^ chelator, and Aβ. The BAPTA-AM and Aβ co-treated group showed lower Ca^2+^ level, followed by reduced ROS level compared to Aβ only treated group (Additional file 5). These results suggest that ROS and Ca^2+^ regulate each other, and this leads to a vicious cycle, which accelerates AD pathology.

### Recovery of Aβ-induced ROS and Ca^2+^ elevation by HDAC6 inhibitor is mediated by acetylation of Prx1

**Fig. 4:**
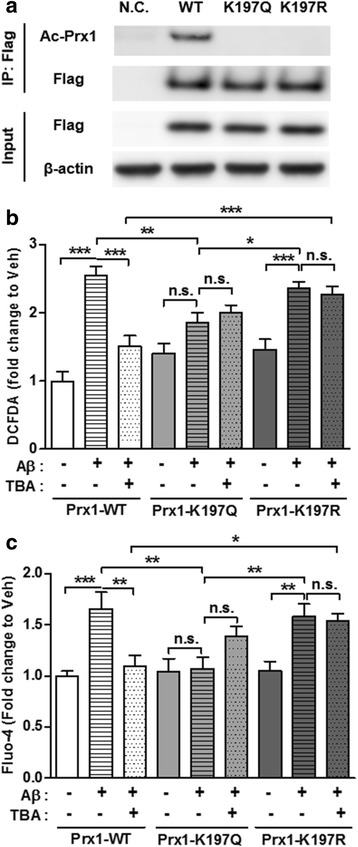
Recovery of Aβ-induced ROS and Ca^2+^ by HDAC6 inhibitor is mediated by acetylation of Prx1. **a** Representative immunoblot showing expression of Prx1-WT or K197Q or K197R-Flag in HT22 cells. Immunoprecipitated exogenous Prx1-Flag showed differential detecting pattern of acetyl-Prx1 among wild-type and mutants Prx1. Exogenous Prx1-Flag was probed by anti-Flag antibody. Beta-actin is loading control. N.C.: Negative Control. **b**, **c** Prx1-WT or K197Q or K197R-Flag transfected HT22 cells were treated with Aβ (2 μM, 24 h) followed by incubated with TBA (0.5 μM, 3 h). Quantitative graph of DCFDA assay for ROS measuring (**b**) and Fluo-4 assay for Ca^2+^ measuring (**c**) showed that acetylation of Prx1 regulates ROS and Ca^2+^ level in the presence of Aβ, respectively. Data are presented as mean ± SEM. (b: *n* = 10, c: *n* = 7, independent experiments) *P < 0.05, **P < 0.01, ***P < 0.001, n.s.: non-significant (two-way ANOVA, Bonferroni post-hoc test)

To determine the role of acetylated Prx1 in the recovery of Aβ-induced ROS and Ca^2+^, we constructed acetyl mimic and silencing mutants, K197Q and K197R respectively, by substituting glutamine (Q) or R for K. The expression level is similar among constructs (Fig. 4a). Previous study shows that acetyl mimic mutant has stronger reducing activity compared to WT [19]. Indeed, acetyl mimic mutant transfected HT22 cells are partially resistant to Aβ-induced ROS elevation, while acetyl silencing mutant shows results similar to WT by Aβ. Moreover, even though TBA was co-treated with Aβ in acetyl silencing mutant transfected HT22 cells, increased ROS level by Aβ was not restored unlike in WT which showed restored ROS levels (Fig. 4b). In terms of Ca^2+^ level, it shows similar patterns to ROS level results (Fig. 4c). Acetyl mimic mutant groups showed protective effects against Aβ-induced increased Ca^2+^ level, and acetyl silencing mutant groups lost their function compared to WT. It provides evidence that increased Ca^2+^ by Aβ could be regulated by ROS level which is modulated by acetyl Prx1. In addition, there were no additive decreasing effects on ROS and Ca2+ levels under Aβ and TBA co-treatment compared to Aβ-only treatment in acetyl mimic mutant groups. This indicates that the ability of TBA to influence Prx1 activity is dependent on acetylation of K197. Thus, these data demonstrate that increased acetylation at K197 of Prx1 by HDAC6 inhibitor contributes to rescue of ROS level followed by rescue of Ca^2+^ level in the presence of Aβ.

### HDAC6 inhibitor rescues mitochondrial axonal transport impaired by Aβ through acetylated Prx1

**Fig. 5:**
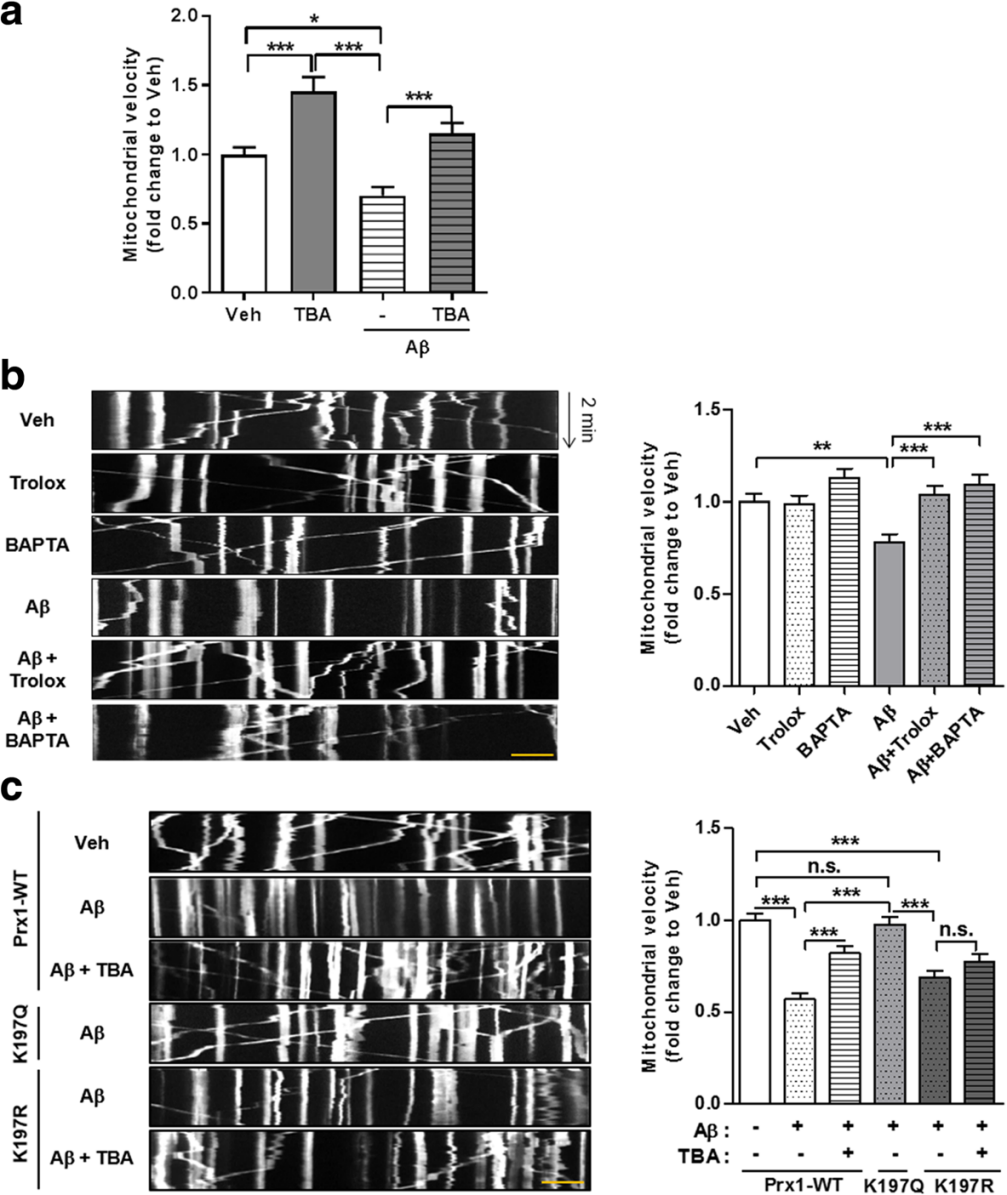
Aβ-induced ROS disrupts mitochondrial axonal transport by elevating Ca^2+^ level. **a** Mitochondrial absolute velocity in axons of primary hippocampal neurons showing TBA recover impaired mitochondrial axonal transport by Aβ. Mito-dsRed2 transfected primary hippocampal neurons were treated with Aβ (2 μM, 24 h) followed by incubated with TBA (0.5 μM, 3 h). Data were obtained from 3 independent experiments (*n* = 30 cells per group). The results were shown as mean ± SEM. *P < 0.05, ***P < 0.001 (two-way ANOVA, Bonferroni post-hoc test) (**b**) Disrupted mitochondrial axonal transport by Aβ is recovered by ROS inhibitor or Ca^2+^ chelator. Mito-dsRed2 transfected primary hippocampal neurons were pretreated with trolox (200 μM) or BAPTA (2 μM) for 1 h before incubation with 2 μM Aβ (24 h). *Left* panel shows kymograph and *right* panel shows quantitative graph of mitochondrial absolute velocity. Data were acquired from 4 independent experiments (*n* = 40 cells per group). The results were shown as mean ± SEM. **P < 0.01, ***P < 0.001 (two-way ANOVA, Bonferroni post-hoc test) **c** Acetylation of Prx1 regulate mitochondrial axonal transport in the presence of Aβ. Prx1-WT or K197Q or K197R and mito-dsRed2 co-transfected primary hippocampal neurons were treated with Aβ (2 μM, 24 h) followed by incubated with TBA (0.5 μM, 3 h). *Left* panel shows kymograph and *right* panel shows quantitative graph of mitochondrial absolute velocity. Data were obtained from 5 independent experiments (*n* = 40 cells per group). The results were shown as mean ± SEM. ***P < 0.001, n.s.: non-significant (one-way ANOVA, Bonferroni post-hoc test), scale bar: 10 μm



It is reported that excessive ROS and Ca^2+^ disrupt axonal transport [14, 15]. Ca^2+^, especially, binds to Miro, which is an adaptor protein that links mitochondria and kinesin, and inhibits mitochondrial binding to kinesin. In addition, our previous reports showed that axonal transport impaired by Aβ is recovered by HDAC6 inhibition [24], which was repeated in Fig. 5a. Thus, we thought that increased ROS and Ca^2+^ are possible mechanisms by which Aβ disrupts axonal transport, and that altered acetylation of Prx1 might be involved in these processes. To determine whether ROS and Ca^2+^ contribute to disrupted axonal transport in the presence of Aβ, we treated Aβ with or without trolox or BAPTA-AM, an ROS inhibitor or Ca^2+^ chelator, respectively, to primary neurons which were transfected pDsRed2-Mito (Fig. 5b and Additional file 6). Consistent with previous reports, the velocity of mitochondrial axonal transport was decreased compared to veh. However, when ROS or Ca^2+^ was blocked in the presence of Aβ, the velocity of mitochondrial axonal transport was recovered. This suggests that Aβ-induced increased ROS and Ca^2+^ cause impairment of mitochondrial axonal transport. Mediated by acetylated Prx1, HDAC6 inhibitor also reduced ROS and Ca^2+^ levels elevated by Aβ. Therefore, we determined whether acetylation of Prx1 also regulates mitochondrial axonal transport, which is downstream of ROS and Ca^2+^, in the presence of Aβ. WT or acetyl mimic (K197Q) or acetyl silencing (K197R) Prx1 mutant transfected primary hippocampal neurons were used for observing mitochondrial axonal transport. Consistent with previous data, Aβ treated WT and acetyl silencing mutant group showed decreased velocity of mitochondrial axonal transport, however, Aβ treated acetyl mimic mutant group still maintained a velocity similar to veh. When the WT and acetyl silencing mutant groups were treated with TBA and Aβ, the velocity of mitochondrial axonal transport was recovered in the WT but not in the acetyl silencing mutant group (Fig. 5c and Additional file 7). These results indicate that acetylation of Prx1, which is modulated by HDAC6 inhibitor, contributes to the recovery of axonal transport impaired by Aβ. Therefore, it is suggested that ROS and Ca^2+^ recovery through increased acetylation of Prx1 is one of the mechanisms by which HDAC6 inhibition rescues axonal transport disrupted by Aβ.

**Additional file 6:** Disrupted mitochondrial axonal transport by Aβ is recovered by ROS inhibitor or Ca^2+^ chelator. Representative mitochondrial movements in axons of primary hippocampal neurons which are related to Fig. 5b. Scale bar: 10 μm. (MOV 574 kb)

**Additional file 7:** Acetylation of Prx1 regulate mitochondrial axonal transport in the presence of Aβ. Representative mitochondrial movements in axons of primary hippocampal neurons which are related to Fig. 5c. Scale bar: 10 μm. (MOV 674 kb)


### HDAC6 inhibitor rescues oxidative stress and mitochondrial transport by elevating acetylation of Prx1 in 5xFAD mice

**Fig. 6:**
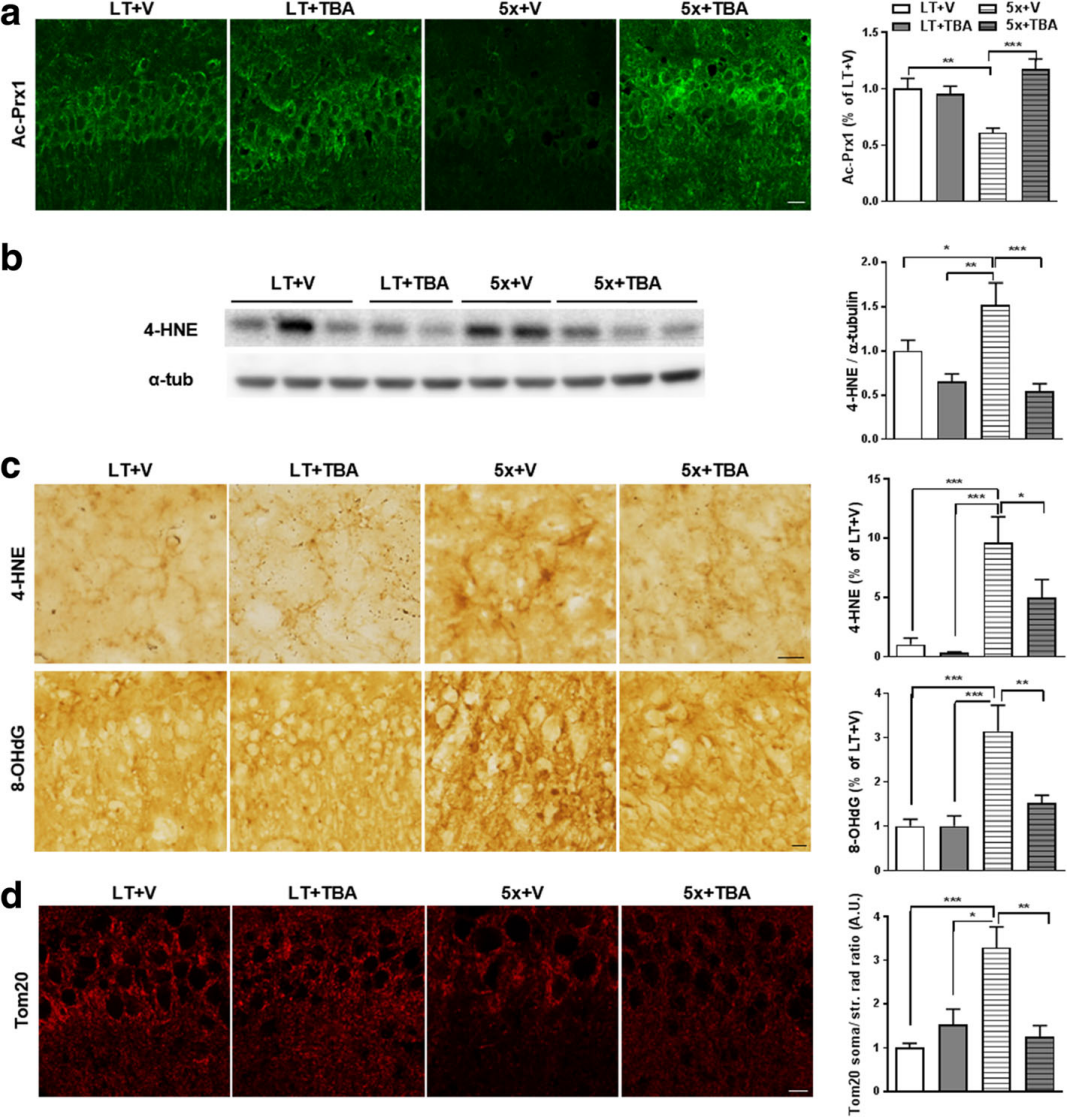
HDAC6 inhibitor rescues oxidative stress and mitochondrial transport by elevating Prx1 acetylation in 5xFAD mice. TBA (100 mg/kg) was i.p. injected to 6-month-old 5xFAD female mice for 4 weeks. Five mice were used in each group. Cryosectioned brain slice were used in (**a**, **c** and **d**). Brain lysates were used in (**b**). **a** Reduced acetylation of Prx1 is recovered by TBA in 5xFAD mice. Representative images of Ac-Prx1 immunoreactivity in CA1 are shown in *left* panel and quantitative graph are in *right* panel. **b**, **c** Oxidative stress is recovered by TBA in 5xFAD mice. Representative immunoblot (*left*) and quantitative analysis (*right*) of 4-HNE are shown in **b**. Representative DAB stained images (*left*) against indicated antibodies and quantitative analysis (*right*) are shown in **c**. Immunoreactivity of 4-HNE and 8-OHdG was quantified in cortex and CA1, respectively. **d** Mitochondrial transport is rescued by TBA in 5xFAD mice. Anti-Tom20 shows mitochondrial distribution in CA1. Representative images are shown in *left* panel. Tom20 immunoreactivity in *right* panel was quantified as the ratio of intensity in soma to that in stratum radiatum. Data are presented as mean ± SEM (*n* = 5 per group). *P < 0.05, **P < 0.01, ***P < 0.001 (two-way ANOVA, Bonferroni post-hoc test), LT: wild-type littermate, 5x: 5xFAD, V: vehicle, TBA: Tubastatin A, scale bar: 10 μm

We demonstrated that increased acetylation of Prx1 using HDAC6 inhibitor has potential therapeutic effects against Aβ by recovering ROS and Ca^2+^ levels, then recovering disrupted mitochondrial axonal transport in cell culture systems. Next, we investigated whether HDAC6 inhibitor also shows these therapeutic effects *in vivo* using 5xFAD mice, an AD model mice. Six-month-old 5xFAD mice were injected TBA (100 mg/kg) for 4 weeks intraperitoneally. Consistent with the data from cultured cells, acetylation of Prx1 was reduced in the brains of 5xFAD mice compared to wild type. However, this reduction was recovered in TBA-injected 5xFAD by showing immunohistochemistry (Fig. 6a). There are reports showing that, in the brains of AD patients, some oxidative stress markers were elevated such as 8-hydroxydeoxyguanosine (8-OHdG), a marker of oxidative damage to DNA and RNA, and 4-hydroxynonenal (4-HNE), a product of lipid peroxidation [31–33]. We observed that 4-HNE and 8-OHdG were also increased in 5xFAD brains by western blotting and immunohistochemistry. However, increased acetylation of Prx1 by TBA recovered 4-HNE and 8-OHdG (Fig. 6b,c). These data suggest that reduced acetylation of Prx1 by Aβ might be involved in increased oxidative stress in 5xFAD. Since we identified that reduced ROS level recovers mitochondrial axonal transport in the presence of Aβ, mitochondrial axonal transport was analyzed with immunohistochemistry using Tom20, a mitochondrial marker protein, in mouse brains as previously described [21] (Fig. 6d). Mitochondrial localization presented by Tom20 immunoreactivity showed more mitochondria accumulation in the somata of neurons in the CA1 region of 5xFAD compared to wild type. However, mitochondria were distributed equally from the somata to the stratum radiatum in CA1 of TBA-injected 5xFAD, indicating that HDAC6 inhibition restores mitochondrial axonal transport *in vivo*. It is suggested that oxidative stress might be involved in the disruption of mitochondrial axonal transport in 5xFAD. Taken together, it is supposed that elevating acetylation of Prx1 by HDAC6 inhibitor plays an important role in recovering oxidative stress, followed by recovering mitochondrial axonal transport *in vivo*. Thus, regulating acetylation of Prx1 using HDAC6 inhibitor could be a new therapeutic strategy in AD.

## Discussion

**Fig. 7:**
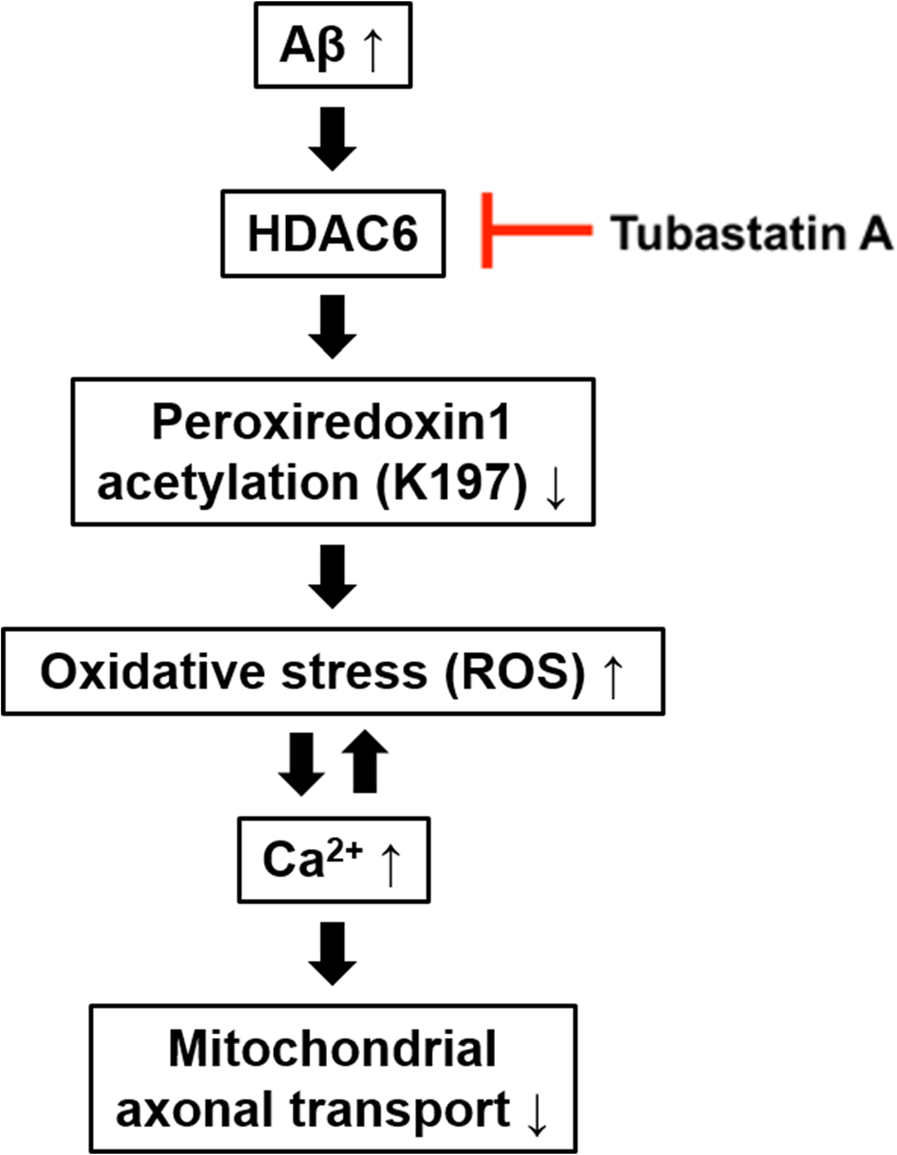
Schematic diagram of the role of HDAC6 inhibitor and acetylation of Prx1 in AD. Increased acetylation of Prx1 by HDAC6 inhibitor rescues impaired mitochondrial axonal transport by restoring oxidative stress followed by reducing Ca^2+^ level in AD

In the present study, we determine the new role of acetylation of Prx1 by HDAC6 modulation in AD pathogenesis related to Aβ. When acetylation of Prx1 at K197 was reduced, mitochondrial axonal transport was disrupted following the elevation of ROS and Ca^2+^ in the presence of Aβ. These pathologic features caused by Aβ were recovered by modulating acetylation of Prx1 through HDAC6 inhibition (Fig. 7). Given that Prx1 is a substrate of HDAC6, which is increased in level and activity in the brains of AD patients [16], reduced acetylation of Prx1 by Aβ might result from HDAC6 overactivation. Since deacetylated Prx1 has decreased anti-oxidant activity [19], excessive ROS and Ca^2+^ caused by Aβ may also be downstream of HDAC6. It is reported that ROS and excessive Ca^2+^ impair mitochondrial axonal transport [14, 15]. Therefore, disrupted mitochondrial axonal transport in the presence of Aβ might be a result of reduced Prx1 acetylation caused by overactivated HDAC6. Previous studies have shown that HDAC6 inhibition rescues the Aβ-induced impairment of mitochondrial axonal transport by increasing acetylation of α-tubulin, resulting in increased microtubule stability and the recruitment of motor proteins to microtubules [3, 24, 34, 35]. Alpha-tubulin is a well-known substrate of HDAC6, and many researches have reported the relationship between acetylated α-tubulin and axonal transport [3, 24, 36, 37]. Here, we additively elucidated a novel mechanism of regulating mitochondrial axonal transport by HDAC6 inhibition, which modulates acetylation of Prx1 thereby regulating ROS and Ca^2+^ levels. This novel mechanism is more important, because it reveals that HDAC6 inhibitor can rescue excessive ROS and Ca^2+^ which are other major cellular pathogenic factors in AD.

In addition, modulation of Prx1 acetylation, not Prx1 itself, is more important for treatment of AD, because Prx1 is already increased in AD patients’ brains [38, 39]. Although researchers consider that this may be the result of a cellular protective mechanism [38, 39], oxidative stress still remain in AD brains. Therefore, increasing acetylation of Prx1 is important to elevate anti-oxidant activity of Prx1. In this study, we demonstrated that elevating acetylation of Prx1 by HDAC6 inhibition in AD condition could recovered Aβ-induced oxidative stress followed by restoring excessive Ca^2+^ and mitochondrial axonal transport. Thus, modulating acetylation of Prx1 could be one of therapeutic targets for AD.

Several researchers showed that reduction or inhibition of HDAC6 ameliorated memory impairment in AD mice model [21–23, 40]. They proposed that the mechanism of memory rescue works by restoring axonal transport by increasing microtubule stability or recovering autophagy. In addition, the novel mechanism presented in this study could explain memory rescue through HDAC6 inhibition. Well-known pathogenic features of Aβ include elevated ROS and disrupted Ca^2+^ homeostasis and axonal transport [1, 41]. Excessive ROS in AD trigger oxidative stress, which leads to neuronal dysfunction and cell death by Aβ production, lipid peroxidation, enhancement of protein oxidation, and disruption of gene regulation through DNA oxidation [8, 31, 42, 43]. Lipid peroxidation causes ion imbalance and impairs metabolism by weakening cell membranes [44]. Moreover, Torres et al. suggested that the severity of cognitive impairment is directly related to the level of oxidative stress [45]. Elevated Ca^2+^ activates several enzymes such as calcineurin and calpain, which leads to neurite atrophy, disruption in synaptic plasticity and apoptotic cell death [12, 13, 46, 47]. A report also shows that the onset of cognitive symptoms in AD is tightly correlated with reduction of Ca^2+^-binding proteins [48, 49]. Since neurons have unique polarized shapes such as long axons, axonal transport is important for communication between cell bodies and axon terminals. Therefore, axonal transport deficit leads to several deleterious effects in neurons. Clearance of misfolded proteins in axons and response to neurotrophic signals or stress insults require axonal transport [3, 5, 7]. Specifically, mitochondrial axonal transport is important for supplying energy to the distal axon to maintain synaptic function [6]. Amyloid precursor protein processing, from which Aβ is generated, is stimulated by axonal blockade [50]. In addition, it is reported that excessive ROS and Ca^2+^ damage axonal transport of mitochondria and synaptic vesicles [14, 15]. These findings suggest that disrupted ROS, Ca^2+^ homeostasis and axonal transport, assumed to be early pathologic events, are crucial for neuronal cell death and cognitive impairment in AD. Therefore, HDAC6 inhibitor could recover memory impairment in AD mice models. Moreover, since disrupted ROS, Ca^2+^ homeostasis and axonal transport are common pathologies of neurodegenerative diseases [3, 13, 51], it is expected that HDAC6 inhibitor could also have therapeutic effects in other neurodegenerative diseases.

To explore potential use for clinical treatment of HDAC6 inhibitor, we tested both posttreatment and pretreatment of TBA (Fig. 2 and Additional file 3). The results were similar in both treatment conditions, in which TBA could recover Aβ-induced ROS and Ca^2+^ levels. This indicates that TBA has both preventive and restorative ability for pathology of AD. However, we observed a little increasing of Ca^2+^ in TBA-alone-pretreatment group unlike in posttreatment group, although it wasn’t severe increase as much as Aβ-alone-treated group.

We showed that ROS can regulate mitochondrial axonal transport mediated by Ca^2+^. There are reports that excessive ROS or Ca^2+^ disrupts mitochondrial axonal transport [14, 15]. However, how ROS alter mitochondrial axonal transport is unclear. Our results show that when Aβ-induced ROS was blocked by ROS inhibitors or increased acetylation of Prx1 by HDAC6 inhibitor, Ca^2+^ level decreased, and rescue of mitochondrial axonal transport followed. It is reported that Ca^2+^ regulates mitochondrial axonal transport by modulating the interaction between mitochondria and motor proteins through Miro, which undergoes conformational changes as Ca^2+^ binds to it [15, 52]. Thus, we revealed for the first time that Ca^2+^ is a key mediator of ROS-induced mitochondrial axonal transport deficit.

Although ROS increase Ca^2+^ level by facilitating Ca^2+^ release from the ER or mitochondria, it is well-known that excessive Ca^2+^ can also elevate ROS by causing mitochondrial dysfunction [13, 31, 53, 54]. Consistent with previous reports, we showed that reduction of Aβ-induced Ca^2+^ by BAPTA decreased ROS levels (Additional file 5). Considering that Aβ-induced ROS elevate Ca^2+^, and that Ca^2+^ also elevates ROS, a vicious cycle is formed. This cyclic nature suggests that mitochondrial axonal transport might be very susceptible to AD.

In this study, we investigated that the elevation of acetylation of Prx1 by HDAC6 inhibitor partially rescues increased ROS level in the presence of Aβ. Only partial recovery was achieved possibly due to other redox regulatory systems, which remain disrupted by Aβ, such as superoxide dismutase-2, glutaredoxin 1 and thioredoxin 1 [55]. The precise mechanism needs to be further studied.

## Conclusions

We demonstrated a novel mechanism: mediated by restoring ROS and Ca^2+^ level in the presence of Aβ, increased acetylation of Prx1 by HDAC6 inhibitor rescues impaired mitochondrial axonal transport. This indicates the new role of Prx1 in regulating mitochondrial axonal transport which is downstream of HDAC6 and the new role of HDAC6 in restoring Aβ-induced oxidative stress and disrupted Ca^2+^ homeostasis. Therefore, HDAC6 inhibition might be a strong therapeutic strategy for AD.

## Additional files

Additional file 1: Human brain samples that were used in the study. F: Female. (PDF 200 kb)
Additional file 2: Anti-acetyl Prx1 (R2-31) antibody specifically detects acetylated Prx1 at K197. R2-31 antibody specificity was validated by immunoprecipitation of Flag tagged Prx1-WT or Prx1-K197R using anti-Flag M2 magnetic beads and probed by R2-31 antibody. Expression and immunoprecipitation of exogenous Prx1-WT-Flag or Prx1-K197R-Flag was confirmed by anti-Flag antibody. Immunoblot of Ac-tub in Input shows TBA works well. N.C.: Negative Control, TBA: Tubastatin A. (PDF 793 kb)
Additional file 3: Pretreatment of TBA also decreases Aβ-induced ROS and Ca^2+^. HT22 cells were pretreated with TBA (0.5 μM) for 1 h before incubation with Aβ (2 μM, 24 h). **a** ROS level was measured by DCFDA assay in HT22 cells. Upper panel is representative images of DCFDA signals (top row) and bright field images (bottom row) and lower panel is quantification of fluorescent intensity (*n* = 6, independent experiments). **b** Ca^2+^ level was measured by Fluo-4 assay in HT22 cells. Left panel is representative images and right panel is quantification of fluorescent intensity (*n* = 4, independent experiments). Data are presented as mean ± SEM. **P* < 0.05, ***P* < 0.01, ****P* < 0.001 (two-way ANOVA, Bonferroni post-hoc test). Scale bar: 100 μm. (PDF 3678 kb)
Additional file 4: NAC, an ROS inhibitor, also can regulate Aβ-induced intracellular calcium level. **a** Aβ-induced ROS was decreased by 2 mM NAC. HT22 cells were pretreated with indicated concentration of NAC for 1 h before incubation with 2 μM Aβ (24 h). Upper panel is representative images of DCFDA signals (top row) and bright field (bottom row) to measure ROS level in HT22 cells and lower penal is quantitative graph (*n* = 5, independent experiments). The results were shown as mean ± SEM. ***P* < 0.01 (one-way ANOVA, Bonferroni post-hoc test) **b** Reduction of Aβ-induced ROS level by NAC can decrease Ca^2+^ level. HT22 cells were pretreated with 2 mM NAC for 1 h before incubation with 2 μM Aβ (24 h). Left panel is representative images of Fluo-4 assay to measure Ca^2+^ level in HT22 cells and right panel is quantitative graph (*n* = 7, independent experiments). The results were shown as mean ± SEM. ***P* < 0.01, ****P* < 0.001 (two-way ANOVA, Bonferroni post-hoc test). Scale bar: 100 μm. (PDF 5662 kb)
Additional file 5: Disrupted Ca^2+^ homeostasis induced by Aβ affects ROS level. **a** Increased Ca^2+^ level induced by Aβ was reduced by 2 μM BAPTA. HT22 cells were pretreated with indicated concentration of BAPTA for 1 h before incubation with 2 μM Aβ (24 h). Left panel is representative images of Fluo-4 assay to measure Ca^2+^ level and quantification is shown in right panel (*n* = 6, independent experiments). The results were shown as mean ± SEM. **P* < 0.05, ***P* < 0.01 (one-way ANOVA, Bonferroni post-hoc test). **b** Reduction of Ca^2+^ by BAPTA can decrease ROS level in the presence of Aβ. HT22 cells were pretreated with 2 μM BAPTA for 1 h before incubation with 2 μM Aβ (24 h). In upper panel, representative DCFDA signals and bright field images were shown in top row and bottom row, respectively. Quantification graph was shown in lower panel (*n* = 6, independent experiments). The results were shown as mean ± SEM. **P* < 0.05, ***P* < 0.01, ****P* < 0.001 (two-way ANOVA, Bonferroni post-hoc test). Scale bar: 100 μm. (PDF 3018 kb)

## Abbreviations

4-HNE: 4-hydroxynonenal
8-OHdG: 8-hydroxydeoxyguanosine
AD: Alzheimer’s disease
Aβ: Beta-amyloid
DAB: 3, 3′-diaminobenzidine
DCFDA: 2′, 7′-dichlorodihydrofluorescein diacetate
DIV: Day in vitro
HDAC6: Histone deacetylase 6
i.p.: Intraperitoneal
NAC: N-acetyl cysteine
PFA: Paraformaldehyde
PMSF: Phenyl-methylsulfonyl fluoride
Prx1: Peroxiredoxin1
PVDF: Polyvinylidene difluoride
ROS: Reactive oxygen species
RT: Room temperature
TBA: Tubastatin A
WT: Wild type
