# Supplementary material for: Increased acetylation of Peroxiredoxin1 by HDAC6 inhibition leads to recovery of Aβ-induced impaired axonal transport
Source: Mol Neurodegener. 2017 Feb 28;12:23. doi: 10.1186/s13024-017-0164-1 (PMC5330132; doi:10.1186/s13024-017-0164-1)
Supplement: Additional file 1: — Human brain samples that were used in the study. F: Female. (PDF 200 kb) [file 13024_2017_164_MOESM1_ESM.pdf]

	Age	Sex	Braack stage
**Normal:**			
1	101	F	I
2	78	F	II
**AD:**			
1	100	F	V
2	79	F	VI
