# Supplementary material for: Increased acetylation of Peroxiredoxin1 by HDAC6 inhibition leads to recovery of Aβ-induced impaired axonal transport
Source: Mol Neurodegener. 2017 Feb 28;12:23. doi: 10.1186/s13024-017-0164-1 (PMC5330132; doi:10.1186/s13024-017-0164-1)
Supplement: Additional file 2: — Anti-acetyl Prx1 (R2-31) antibody specifically detects acetylated Prx1 at K197. R2-31 antibody specificity was validated by immunoprecipitation of Flag tagged Prx1-WT or Prx1-K197R using anti-Flag M2 magnetic beads and probed by R2-31 antibody. Expression and immunoprecipitation of exogenous Prx1-WT-Flag or Prx1-K197R-Flag was confirmed by anti-Flag antibody. Immunoblot of Ac-tub in Input shows TBA works well. N.C.: Negative Control, TBA: Tubastatin A. (PDF 793 kb) [file 13024_2017_164_MOESM2_ESM.pdf]

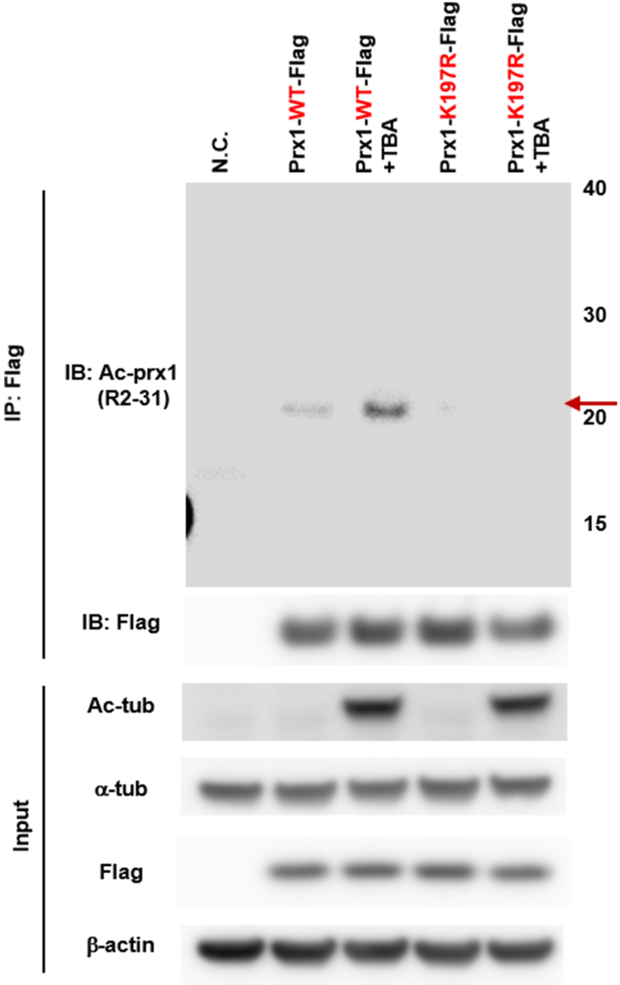

N.C.
Prx1-WT-Flag
Prx1-WT-Flag +TBA
Prx1-K197R-Flag
Prx1-K197R-Flag +TBA
40
30
20
15
IP: Flag
IB: Ac-prx1 (R2-31)
IB: Flag
Input
Ac-tub
α-tub
Flag
β-actin
