# Supplementary figures and images for: Increased acetylation of Peroxiredoxin1 by HDAC6 inhibition leads to recovery of Aβ-induced impaired axonal transport
Source: Mol Neurodegener. 2017 Feb 28;12:23. doi: 10.1186/s13024-017-0164-1 (PMC5330132; doi:10.1186/s13024-017-0164-1)

**a**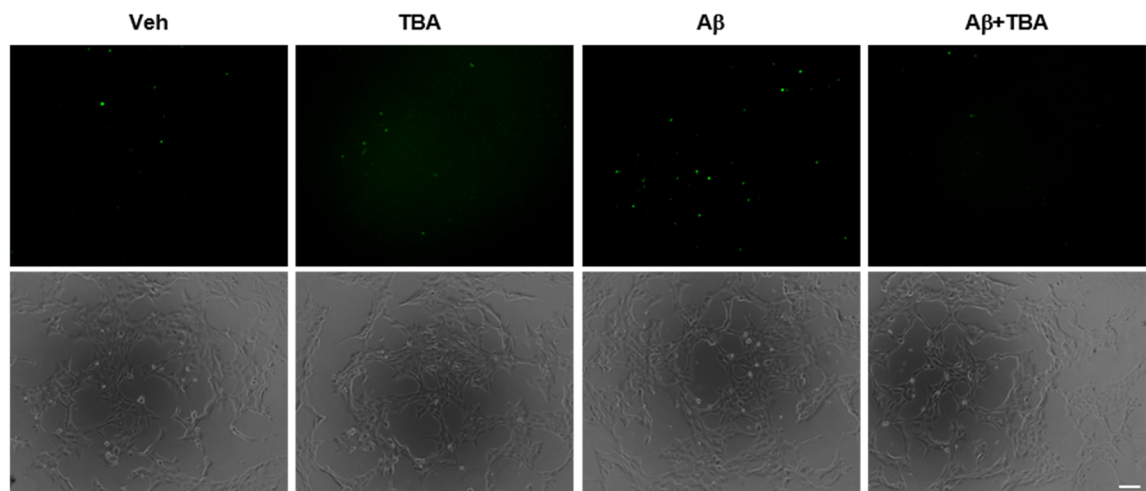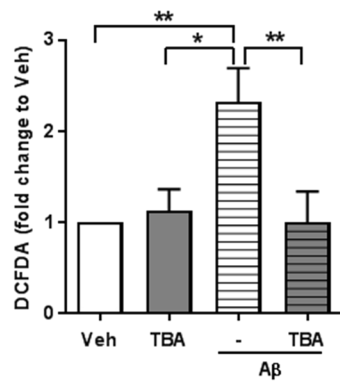**b**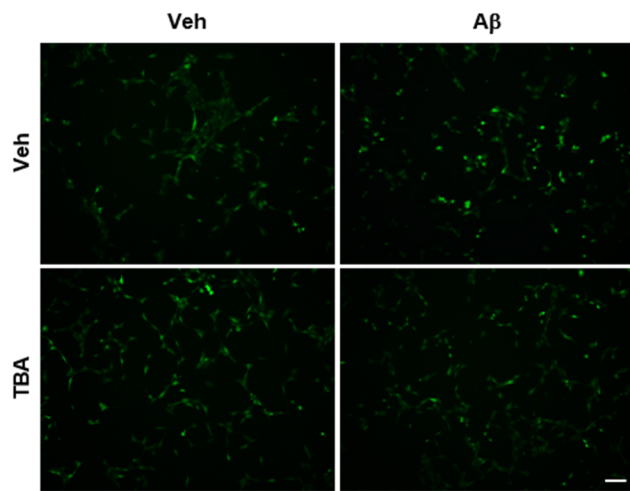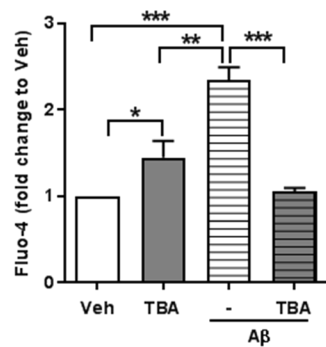

Supplement: Additional file 3: — Pretreatment of TBA also decreases Aβ-induced ROS and Ca2+. HT22 cells were pretreated with TBA (0.5 μM) for 1 h before incubation with Aβ (2 μM, 24 h). a ROS level was measured by DCFDA assay in HT22 cells. Upper panel is representative images of DCFDA signals (top row) and bright field images (bottom row) and lower panel is quantification of fluorescent intensity (n = 6, independent experiments). b Ca2+ level was measured by Fluo-4 assay in HT22 cells. Left panel is representative images and right panel is quantification of fluorescent intensity (n = 4, independent experiments). Data are presented as mean ± SEM. *P < 0.05, **P < 0.01, ***P < 0.001 (two-way ANOVA, Bonferroni post-hoc test). Scale bar: 100 μm. (PDF 3678 kb) [file 13024_2017_164_MOESM3_ESM.pdf]

**a**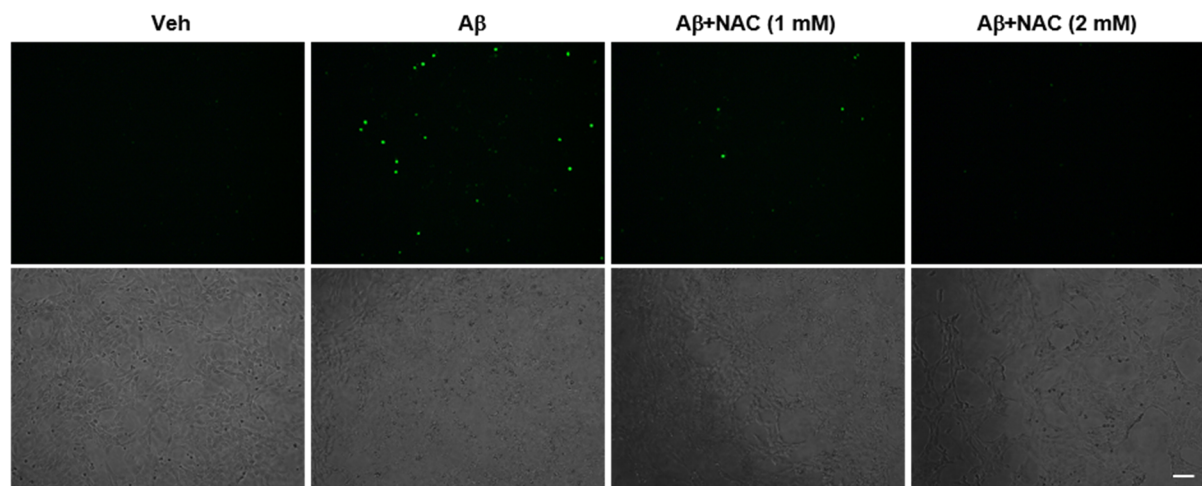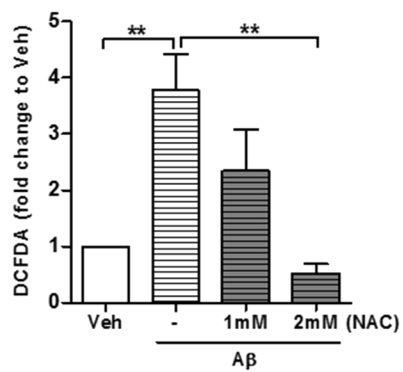**b**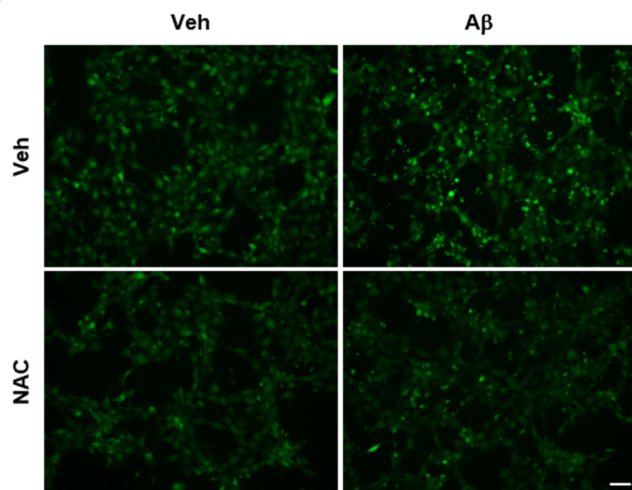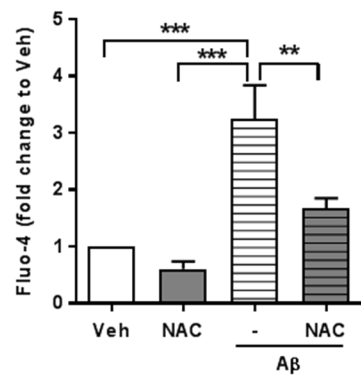

Supplement: Additional file 4: — NAC, an ROS inhibitor, also can regulate Aβ-induced intracellular calcium level. a Aβ-induced ROS was decreased by 2 mM NAC. HT22 cells were pretreated with indicated concentration of NAC for 1 h before incubation with 2 μM Aβ (24 h). Upper panel is representative images of DCFDA signals (top row) and bright field (bottom row) to measure ROS level in HT22 cells and lower penal is quantitative graph (n = 5, independent experiments). The results were shown as mean ± SEM. **P < 0.01 (one-way ANOVA, Bonferroni post-hoc test) b Reduction of Aβ-induced ROS level by NAC can decrease Ca2+ level. HT22 cells were pretreated with 2 mM NAC for 1 h before incubation with 2 μM Aβ (24 h). Left panel is representative images of Fluo-4 assay to measure Ca2+ level in HT22 cells and right panel is quantitative graph (n = 7, independent experiments). The results were shown as mean ± SEM. **P < 0.01, ***P < 0.001 (two-way ANOVA, Bonferroni post-hoc test). Scale bar: 100 μm. (PDF 5662 kb) [file 13024_2017_164_MOESM4_ESM.pdf]

a

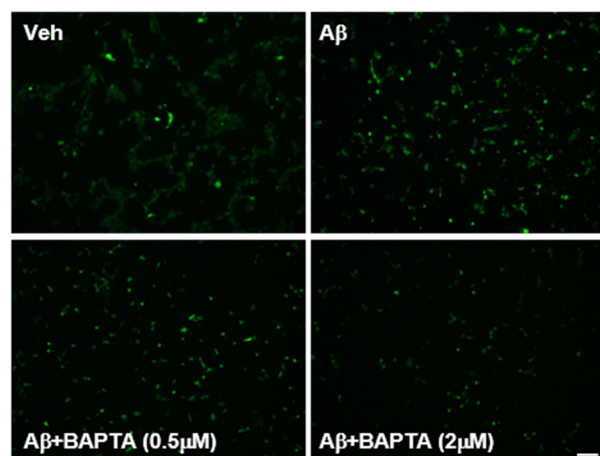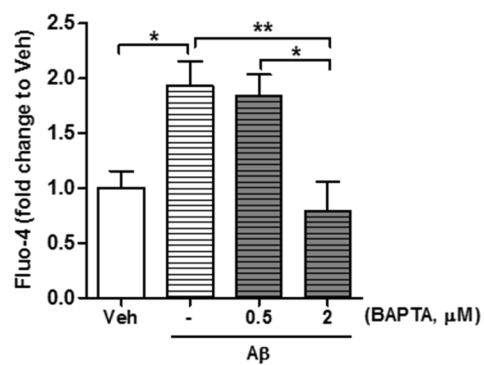

b

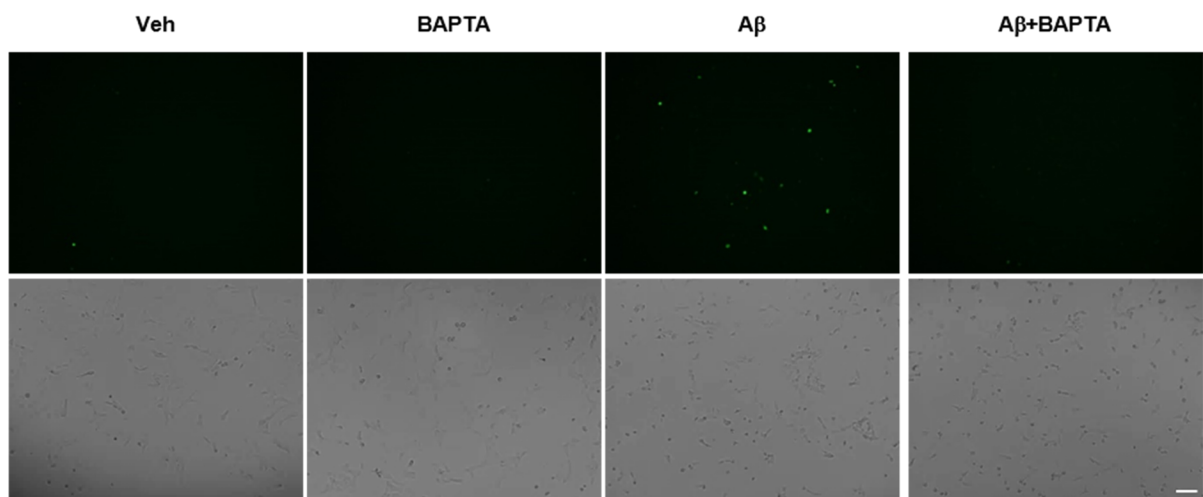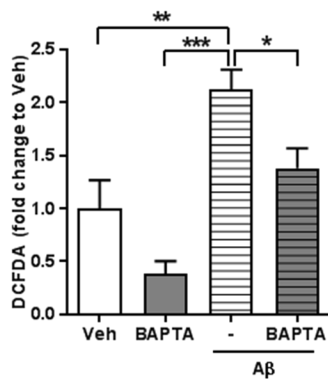

Supplement: Additional file 5: — Disrupted Ca2+ homeostasis induced by Aβ affects ROS level. a Increased Ca2+ level induced by Aβ was reduced by 2 μM BAPTA. HT22 cells were pretreated with indicated concentration of BAPTA for 1 h before incubation with 2 μM Aβ (24 h). Left panel is representative images of Fluo-4 assay to measure Ca2+ level and quantification is shown in right panel (n = 6, independent experiments). The results were shown as mean ± SEM. *P < 0.05, **P < 0.01 (one-way ANOVA, Bonferroni post-hoc test). b Reduction of Ca2+ by BAPTA can decrease ROS level in the presence of Aβ. HT22 cells were pretreated with 2 μM BAPTA for 1 h before incubation with 2 μM Aβ (24 h). In upper panel, representative DCFDA signals and bright field images were shown in top row and bottom row, respectively. Quantification graph was shown in lower panel (n = 6, independent experiments). The results were shown as mean ± SEM. *P < 0.05, **P < 0.01, ***P < 0.001 (two-way ANOVA, Bonferroni post-hoc test). Scale bar: 100 μm. (PDF 3018 kb) [file 13024_2017_164_MOESM5_ESM.pdf]
